# Upper gastrointestinal alterations in kidney transplant candidates

**DOI:** 10.1590/2175-8239-JBN-3829

**Published:** 2018-05-14

**Authors:** João Pedro Homse, João Pedro Sant'Anna Pinheiro, Mariana Lopes Ferrari, Mirella Tizziani Soares, Rogério Augusto Gomes Silveira, Mariana Espiga Maioli, Vinicius Daher Alvares Delfino

**Affiliations:** 1 Pontifícia Universidade Católica do Paraná, Londrina, PR, Brasil.; 2 Universidade Estadual de Londrina, Londrina, PR, Brasil.

**Keywords:** kidney transplantation, kidney failure, chronic, renal dialysis, endoscopy, digestive system, Helicobacter pylori, insuficiência renal crônica, transplante de rim, diálise renal, endoscopia do sistema digestório, Helicobacter pylori

## Abstract

**Introduction:**

The incidence of gastrointestinal disorders among patients with chronic kidney disease (CKD) is high, despite the lack of a good correlation between endoscopic findings and symptoms. Many services thus perform upper gastrointestinal (UGI) endoscopy on kidney transplant candidates.

**Objectives:**

This study aims to describe the alterations seen on the upper endoscopies of 96 kidney-transplant candidates seen from 2014 to 2015.

**Methods:**

Ninety-six CKD patients underwent upper endoscopic examination as part of the preparation to receive kidney grafts. The data collected from the patients' medical records were charted on Microsoft Office Excel 2016 and presented descriptively. Mean values, medians, interquartile ranges and 95% confidence intervals of the clinic and epidemiological variables were calculated. Possible associations between endoscopic findings and infection by *H. pylori* were studied.

**Results:**

Males accounted for 54.17% of the 96 patients included in the study. Median age and time on dialysis were 50 years and 50 months, respectively. The most frequent upper endoscopy finding was enanthematous pangastritis (57.30%), followed by erosive esophagitis (30.20%). Gastric intestinal metaplasia and peptic ulcer were found in 8.33% and 7.30% of the patients, respectively. *H. pylori* tests were positive in 49 patients, and *H. pylori* infection was correlated only with non-erosive esophagitis (P = 0.046).

**Conclusion:**

Abnormal upper endoscopy findings were detected in all studied patients. This study suggested that upper endoscopy is a valid procedure for kidney transplant candidates. However, prospective studies are needed to shed more light on this matter.

## RESUMO

**Introdução:**

A incidência de doenças gastrointestinais altas em pacientes com doença renal crônica é elevada, porém não há boa correlação entre achados endoscópicos e sintomas. Assim, muitos serviços preconizam a realização de Endoscopia Digestiva Alta (EDA) nos candidatos a transplante renal.

**Objetivos:**

Descrever alterações endoscópicas presentes em 96 candidatos a transplante renal no período de 2014 a 2015.

**Métodos:**

Noventa e seis pacientes renais crônicos submetidos à EDA como preparo para transplante renal. Prontuários médicos dos pacientes foram revisados, os dados tabulados no programa Microsoft Office Excel 2016 e apresentados de maneira descritiva. Calculou-se média, mediana, intervalo interquartílico e intervalo de confiança de 95% das variáveis utilizadas. Alterações endoscópicas foram apresentadas quanto ao número, intervalo de confiança e valor de P, e correlacionadas com a presença ou ausência de infecção por *Helicobacter pylori*.

**Resultados:**

Dos 96 pacientes, 54,17% eram homens e 45,83% mulheres. As medianas de idade e tempo em diálise foram 50 anos e 50 meses, respectivamente. O achado mais comum na EDA foi pangastrite enantematosa (57,30%), seguida de esofagite erosiva (30,20%). Metaplasia intestinal gástrica e úlcera péptica foram encontradas em 8,33% e 7,30% dos pacientes, respectivamente. Pesquisa para *H. pylori* foi positiva em 49 pacientes, e somente houve correlação entre infecção por *H. pylori* e esofagite não erosiva (P = 0,046).

**Conclusão:**

Afecções gastrointestinais foram detectadas em todos os pacientes estudados. Os achados deste estudo sugerem que a realização de EDA em candidatos a receber transplante renal é desejável. Entretanto, estudos prospectivos são necessários para responder a esta questão.

## INTRODUCTION

Managing patients with chronic kidney disease (CKD) can be challenging on account of the significant number of comorbidities they present, which include diseases of the upper gastrointestinal (UGI) tract often undetected due to the poor correlation between clinical findings and diagnostic tests.^1^ Therefore, screening endoscopic examination is needed to catch UGI diseases in patients with CKD.

Recent studies on the topic have reported that the more common UGI diseases to affect patients with CKD are gastritis; erosive duodenitis and esophagitis; peptic ulcers; infection by *Helicobacter pylori* (*H. pylori);* and bleeding (possibly fatal, since patients with CKD are often on antiplatelet and anticoagulant medication).^2^ The pathophysiology of UGI injuries is complex and stems, among other things, from the use of medication to deal with the comorbidities and metabolic alterations arising from CKD; increased gastrin and ammonia serum levels; gastroesophageal reflux disease; and infection by *H. pylori.* 1 ^,^ 2

A review available on UpToDate^3^ reported lower incidences of *H. pylori* in patients on dialysis than in patients with early-stage disease. Angiodysplasia and erosive esophagitis are more frequently seen in patients with CKD than in the general population.

Proper patient management requires the recognition of diseases of the UGI tract. This requirement becomes even more important for kidney transplant candidates receiving grafts from living or deceased donors, since they are likely to undergo large surgery and the related significant metabolic and psychological distress. High-dose corticosteroid therapy places kidney transplant candidates at higher risk of UGI tract complications.^4^ Immunosuppressant therapy prescribed after successful renal transplantation also increases the risk of UGI diseases. Although recommended by many authors,^2^^,^ 4 there is no consensus over the use of upper endoscopy in the assessment of kidney transplant candidates; nonetheless, the procedure is part of the protocol for kidney transplant recipients at Hospital Evangélico de Londrina (HEL).

The high incidence of GI disorders in kidney transplant candidates is well documented in the literature.^1^^,^ 2 ^,^ 5 However, there is an absolute lack of Brazilian data on the matter. Therefore, this study aimed to describe UGI tract alterations in kidney transplant candidates with CKD to check whether upper endoscopy should be performed routinely as part of patient assessment.

## METHODS

This cross-sectional study enrolled 96 patients with chronic kidney disease aged 18 or older treated in dialysis units registered with the kidney transplant service at HEL in 2014 and 2015 (three dialysis units in the Londrina, two in Cornélio Procópio, one in Apucarana, and one in Santo Antônio da Platina in Northern Paraná); the patients included in the study underwent upper endoscopy as part of the protocol devised by the service for kidney transplant recipients. The Review Board of Irmandade da Santa Casa de Londrina approved the study (CAAE: 57099816.1.0000.0099).

Upper endoscopies were performed in an outpatient setting by two physicians from the gastroenterology/endoscopy service at HEL. Histological identification of infection by *H. pylori* was performed systematically based on biopsy specimens taken from the gastric antrum and other areas of the stomach of the patients when clinically indicated. The specimens were fixed on 10% formalin and processed using the Giemsa stain. Cases of gastritis were categorized based on the Sidney System^6^ (topography: pangastritis, body gastritis, and antral gastritis; category: enanthematous, deep and superficial erosive, atrophic, hemorrhagic gastritis, reflux, hyperplastic mucosal folds; grade: mild, moderate, and severe). Cases of esophagitis were categorized based on the Los Angeles Classification System^7^ (grades A, B, C or D) and on the Savary-Miller^7^ classification (grades I, II, III or IV). The patients were also examined for hiatal hernia, peptic ulcer (Sakita Classification System^8^ - A, H or S) and duodenitis (enanthematous and erosive). The physicians performing the endoscopic examinations produced the endoscopy reports. Clinical, workup, and demographic data and information such as time on dialysis, comorbidities, and prescribed medication were collected from the patients' medical records.

The collected data were processed using Microsoft Office Excel 2016 and presented descriptively.

The Shapiro-Wilk test was used to analyze the normality of the clinical epidemiological data; normally distributed data had *p* > 0.05, whereas data not following a normal distribution had *p* < 0.05. Mean values and the 95% confidence interval (95% CI) were calculated for samples with a normal distribution; median values and interquartile ranges (IQR) were calculated for samples not following a normal distribution.

Alterations observed in endoscopic examination were analyzed for their number (n), 95% CI, and *p*-value, and were assessed for possible correlations with infection by *H. pylori* based on the chi-squared test or Fisher's exact test.

## OBJECTIVES

The primary goal of the study was to describe the alterations seen in the upper endoscopies of 96 kidney transplant candidates performed in 2014 and 2015; the secondary goal was to assess the prevalence of infection by *H. pylori*.

## RESULTS

Table 1 shows the results derived from the analysis of the data collected from the charts and endoscopy reports of the 96 kidney transplant candidates included in the study registered with the Instituto do Rim de Londrina and Hospital Evangélico de Londrina.

**Table 1:** Clinical variables and the respective mean values, medians, interquartile ranges (IQR), and 95% confidence intervals (95% CI) observed in 96 kidney transplant candidates submitted to upper endoscopy

Variable	Median or mean value	IQR or 95% CI	P
Age (years)†	50	41 – 58.5	
Sex N (%)	M: 48 (57.14%)		
	F: 36 (42.86%)		*P = 0.230‡*
Race/Skin color	W: 58 (69.05%)		
	B: 12 (14.29%)		
	Br: 14 (16.67%)		*P < 0.001#*
Height (meters)ỻ	1.65	1.63 – 1.67	
BMI (Kg/m^2^) †	24.22	21.48 – 26.86	
Albumin (g/dL)†	3.8	3.5 – 4.2	
Hb (g/dL)ỻ	12.01	11.69 – 12.52	
Time on Dialysis (months)†	50	23 – 93.5	

† Data presented as mean value and 95% CI;

ỻ Data presented as medians and IQR; BMI (body mass index); Hb (hemoglobin); W (white), B (black), Br (brown);

‡ Test of proportion (1 sample);

# Test of proportion.

The upper endoscopy results of the 96 patients revealed that everyone had some form of UGI tract alteration. Table 2 shows that the number of alterations identified was larger than the number of patients (96), as many had more than one alteration.

**Table 2:** Upper endoscopy alterations of 96 kidney transplant candidates with chronic kidney disease

UGI alterations	N (%)
EE	29 (30.2%)
nEE	22 (22.9%)
HH	27 (28.1%)
ErG	19 (19.8%)
EnG	18 (18.8%)
ErPG	7 (7.3%)
EnPG	55 (57.3%)
PU	7 (7.3%)
ErD	24 (25%)
EnD	23 (24%)

* Classification according to the Sidney,^6^ Los Angeles,^7^ Savary-Miller^7^ and Sakita^8^ Systems. * EE (erosive esophagitis), nEE (non-erosive esophagitis), HH (hiatal hernia), PU (peptic ulcer), ErG (erosive gastritis), EnG (enanthematous gastritis), ErPG (erosive pangastritis), EnPG (enanthematous pangastritis), ErD (erosive duodenitis), EnD (enanthematous duodenitis).

Eighty-four of the 96 patients were tested for infection by *H. pylori* (the physicians performing the endoscopic examinations cleared 12 patients from additional testing); 82 patients had tissue samples removed during upper endoscopy; and the rapid urease test was performed in two patients. Forty-nine patients tested positive (58.33%) - 47 from histology and two from urease tests.

Table 3 describes the alterations and their association with infection by *H. pylori*.

**Table 3:** Association between alterations found in upper endoscopy and presence of *H. pylori* in 84 kidney transplant candidates with chronic kidney disease

Upper endoscopy finding	Upper endoscopy findings N	Presence of *H. pylori* N (%)	Absence of *H. pylori* N (%)	P
EE (+)	25	13 (52.0%)	12 (48.0%)	0.6
EE (-)	59	36 (61.0%)	23 (39.0%)	
nEE (+)	20	16 (80.0%)	4 (20.0%)	0.046
nEE (-)	64	33 (51.6%)	31 (48.4%)	
HH (+)	24	14 (58.3%)	10 (41.7%)	1
HH (-)	60	35 (58.3%)	25 (41.7%)	
ErG (+)	17	9 (52.9%)	8 (47.1%)	0.818
ErG (-)	67	40 (59.7%)	27 (40.3%)	
EnG (+)	13	5 (38.5%)	8 (61.5%)	0.202
EnG (-)	71	44 (62.0%)	27 (38.0%)	
ErPG (+)	7	6 (85.7%)	1 (2.8%)	0.230
ErPG (-)	77	43 (55.8%)	34 (44.2%)	
EnPG (+)	51	30 (58.8%)	21 (41.2%)	1
EnPG (-)	33	19 (57.6%)	14 (42.4%)	
PU (+)	7	6 (85.7%)	1 (14.3%)	0.230
PU (-)	77	43 (55.8%)	34 (44.2%)	
ErD (+)	23	14 (60.9%)	9 (39.1%)	0.967
ErD (-)	61	35 (57.4%)	26 (42.6%)	
EnD (+)	21	10 (47.6%)	11 (52.4%)	0.3711
EnD (-)	49	39 (61.9%)	24 (38.1%)	

(+): finding present, (-): finding absent, EE (erosive esophagitis), nEE (non-erosive esophagitis), HH (hiatal hernia), PU (peptic ulcer), ErG (erosive gastritis), EnG (enanthematous gastritis), ErPG (erosive pangastritis), EnPG (enanthematous pangastritis), ErD (erosive duodenitis), EnD (enanthematous duodenitis).

Intestinal metaplasia - a histological finding - is not shown in Table 3, although it was present in the biopsies of seven of 84 patients (8.33%); *H. pylori* was found in five of them (*p* = 0.6937; chi-squared test).

As mentioned above, 12 patients were cleared from additional testing for *H. pylori*. The upper endoscopy findings of these patients were similar to the findings observed in the other 84 patients tested for *H. pylori*: EE in four patients; nEE in 2; HH in 3; ErG in 2; EnG in 5; ErPG: in none; EnPG in 4; PU: in none; ErD in 1; and EnD in two.

## DISCUSSION

Patients on dialysis are often affected by UGI tract diseases.^1^^,^ 2 ^,^ 5 Given the lack of studies on this topic in Brazil, this study aimed to look into these conditions and verify the validity of routinely carrying out upper endoscopies in kidney transplant candidates at the HEL Transplant Service.

Upper endoscopy revealed that 100% of the patients had some form of UGI tract alteration, many of which potentially severe, including peptic ulcers, gastritis, erosive duodenitis, and intestinal metaplasia. Gastric intestinal metaplasia is known to precede dysplasia in the cascade of precancerous gastric lesions.^9^ Although not categorized as complete or incomplete - the latter type associated with precancerous gastric lesions,^10^ seven patients had intestinal metaplasia, indicating a greater need to follow individuals with CKD using upper endoscopy to detect early cases of gastric cancer, particularly when other predisposing factors are present.^9^^,^ 11 These recommendations are prescribed to individuals from the general population with gastric intestinal metaplasia. To our knowledge, there is no specific recommendation to follow subjects with CKD or kidney transplant patients with upper endoscopy.

Since this was a retrospective study, we were unable to establish correlations between the digestive symptoms presented by the patients. However, most of the patients did not complain of symptoms that would otherwise call for examination by upper endoscopy, and the procedures were carried out as part of the protocol in place at the service. We believe this study warranted the routine use of upper endoscopy in the preparation of patients with CKD for kidney transplantation.

Tests for *H. pylori and the analysis of various upper endoscopy findings* revealed significant presence of the bacteria only in cases of non-erosive esophagitis (*p* = 0.046; chi-squared test). Although chronic infection by *H. pylori* has been correlated with the onset of peptic ulcers, an association between the two was not found in our study, possibly on account of the small sample size; nonetheless, six of the seven documented cases were infected with *H. pylori*. Possibly for the same reason we were unable to find a statistically significant association between the presence of *H. pylori* and intestinal metaplasia (in which chronic infection by *H. pylori* has also been implicated), although five of the seven patients were histologically positive for *H. pylori*.

The retrospective nature of this study is one of its limitations, in addition to the absence of correlations with clinical symptoms, the relatively small number of enrolled patients (96), and the fact that 12 patients were not tested for *H. pylori*.

Bacci et al. (2014) examined endoscopic alterations in patients on dialysis and reported antral gastritis as the main finding, followed by erosive duodenitis.^12^ Sibinović et al. (2006) also described some pathologic alterations, including erosion, angiodysplasia, ulcers, and polyps. As mentioned in this study and suggested in the literature, upper endoscopy should be offered to patients on dialysis and particularly to kidney transplant candidates as a means to prevent possible postoperative complications.^2^

Krishnan et al. (2011) described the following endoscopic findings in kidney transplant candidates: esophagitis, gastritis, and antral erosion. In the second stage of the study, the authors divided uremic patients based on whether they were infected or not by *H. pylori* and compared the groups for endoscopic findings, time on dialysis, and GI symptoms. The comparisons did not elicit statistically significant differences.^1^ Significant UGI alterations were found in asymptomatic patients, as also reported by Sotoudehmanesh et al. (2003), in a study in which no significant associations were found between symptoms and gastrointestinal lesions in hemodialysis patients.^13^

Patients are prescribed immunosuppressants to treat possible cases of graft rejection after transplantation. The prescribed medications include cyclosporine, tacrolimus, mycophenolate mofetil (MMF), and prednisone.^14^ Proton pump inhibitors (PPIs) and H2 antagonists are frequently used to treat UGI tract diseases. The possible interactions between immunosuppressants and drugs used to treat gastrointestinal diseases must be considered. Omeprazole may enhance the effect of tacrolimus, since it affects the liver metabolism of enzyme CYP2C19; patients concomitantly on both drugs must be strictly monitored. Omeprazole also reduces the effect of MMF therapy by increasing gastric pH levels. Caution is needed when ranitidine and cyclosporine are prescribed together, because of the negative association produced by the two drugs. The risk of these interactions may be decreased if upper endoscopy is performed in all kidney transplant candidates to identify lesions and introduce early therapy.

Dobies et al. (2016) looked into the occurrence of gastrointestinal tract diseases seen in endoscopy and colonoscopy examinations of patients submitted to kidney transplantation. The findings of the 45 patients the authors analyzed were similar to the findings of the patients enrolled in our study, i.e., the vast majority had gastrointestinal lesions (eight patient had esophagitis; 33 had gastritis or duodenitis; three had gastric polyps, seven had non-specific colon inflammation; two had colorectal cancer; and 14 had colon polyps).^15^ Teenan et al. (1993) also examined transplant patients using endoscopy two and four months after surgery. Sixteen of the 33 patients included in their study had duodenitis; ten had gastritis; and four had gastric ulcers.^16^ Ponticelli and Passerini (2005) noted that the more common gastrointestinal complications were oral lesions, esophagitis, and peptic ulcers.^17^ The authors added that the frequency of ulcers had diminished substantially in their service since patients began to be actively selected for evidence of peptic ulcers before transplantation. The immediate postoperative care of transplant patients coincides with a critical moment for the onset of new lesions; therefore, patients have to be closely followed for the first few months after surgery.^2^^,^ 8 ^,^ 17

Numerous factors may contribute with the development of peptic ulcers after transplantation. An important risk factor, in addition to history of ulcers, is colonization by *H. pylori.* 4 ^,^ 14 ^,^ 17

Infection by *H. pylori* has been described in more than 90% of the cases of gastric MALT (mucosa associated lymphoid tissue) lymphoma, as reported by Bayerdorffer et al. (1995). The eradication of *H. pylori* has led to the decline or regression of gastric lesions caused by low-grade B-cell MALT lymphomas, suggesting that patients infected by *H. pylori* should be treated.^18^

Shousha et al. (1990) compared the presence of infection by *H. pylori* in patients with end-stage renal disease and dyspeptic controls without CKD. Interestingly, a smaller proportion of the individuals in the group with CKD had *H. pylori* infection (12.24% vs. 51.42% in the control group). The authors were unable to explain why they found these results, but attributed the finding to the use of medication by the group with CKD.^19^ Using a similar method, Ozgür et al. (1997) found that patients on hemodialysis were not protected against infection by *H. pylori*. The infection rate among patients on HD was 60%, whereas 70% of the kidney transplant candidates had infection by *H. pylori*. The prevalence among controls was 64%.^20^ These findings were similar to what was observed in our study, in which infection by *H. pylori* was found in 58% of the patients.

As a result of being positively associated with peptic ulcers, infection by *H. pylori* might generate postoperative complications in kidney transplant patients. The therapy to eradicate *H. pylori* given to patients with CKD might be similar to the treatment offered to disease-free individuals. One of the schemes consists of amoxicillin, clarithromycin, and PPIs (Omeprazole) for seven days. However, the drug interactions between the medications used to treat *H. pylori* and the immunosuppressants given to kidney transplant patients must be considered. Clarithromycin enhances the effect of tacrolimus, since it affects the liver/intestinal metabolism of enzyme CYP3A4; therefore, this drug combination should be avoided. The combined administration of amoxicillin and mycophenolate mofetil (MMF) should also be avoided, since the first decreases the effect of the latter.^14^ The relevance of performing upper endoscopies and biopsies to identify lesions and offer early treatment to transplant candidates with *H. pylori* and thus avoid postoperative complications is again reinforced.

Patients with CKD on pre-dialysis care, peritoneal dialysis (PD), and hemodialysis (HD) have significantly more upper gastrointestinal bleeding (UGIB) events than the general population (HD > PD > pre-dialysis care).^21^^,^ 22 In addition to other factors connected to uremia, the higher incidence of UGIB among patients on HD is partly explained by the repeated use of heparin in HD sessions.^21^ Kidney transplant patients also have a higher incidence of UGIB than the general population.^23^ Therefore, these patients have to be closely monitored for the possibility of UGIB. We were unable to find comparisons between rates of peptic ulcers/UGIB in these patients and individuals on HD, PD or pre-dialysis care.

## CONCLUSION

Successful kidney transplantation requires the monitoring and elimination of possible pre and post operative risks. UGI disorders cause significant morbidity and are highly prevalent in this scenario - every patient enrolled in this study was found to have upper digestive endoscopy alterations, including peptic ulcers and gastric intestinal metaplasia. With that in mind, upper digestive endoscopies might be beneficial to kidney transplant candidates, regardless of symptomatology presented. However, only prospective studies will be sufficient to recomend this procedure as a routine examination to be incorporated into all renal transplant services.
